# Formulaire Des Médicaments Nouveaux Pour 1899

**Published:** 1899-04

**Authors:** 


FORMULAIRE DES MÉDICAMENTS NOUVEAUX POUR 1899. Par H. BOCQUILLON-LIMOUSIN, pharmacien de 1re classe, lauréat de l'École de pharmacie de Paris: Introduction par le Dr. Huchard, médecin des hôpitaux. 1 vol. in-18 de 324 pages, cartonné. Paris: Librairie J. B. Baillière et Fils. 1899.

The tenth edition of this popular little work needs no introduction to the medical profession, its success speaks for itself. It is brought down to the present by the introduction of such drugs and remedial measures as have been admitted to modern scientific therapy.

